# Supplementary material for: Quorum sensing orchestrates parallel cell death pathways in Vibrio cholerae via Type 6 secretion-dependent and -independent mechanisms
Source: Proc Natl Acad Sci U S A. 2024 Nov 5;121(46):e2412642121. doi: 10.1073/pnas.2412642121 (PMC11573629; doi:10.1073/pnas.2412642121)
Supplement: Supplementary file 1 — Appendix 01 (PDF) [file pnas.2412642121.sapp.pdf]

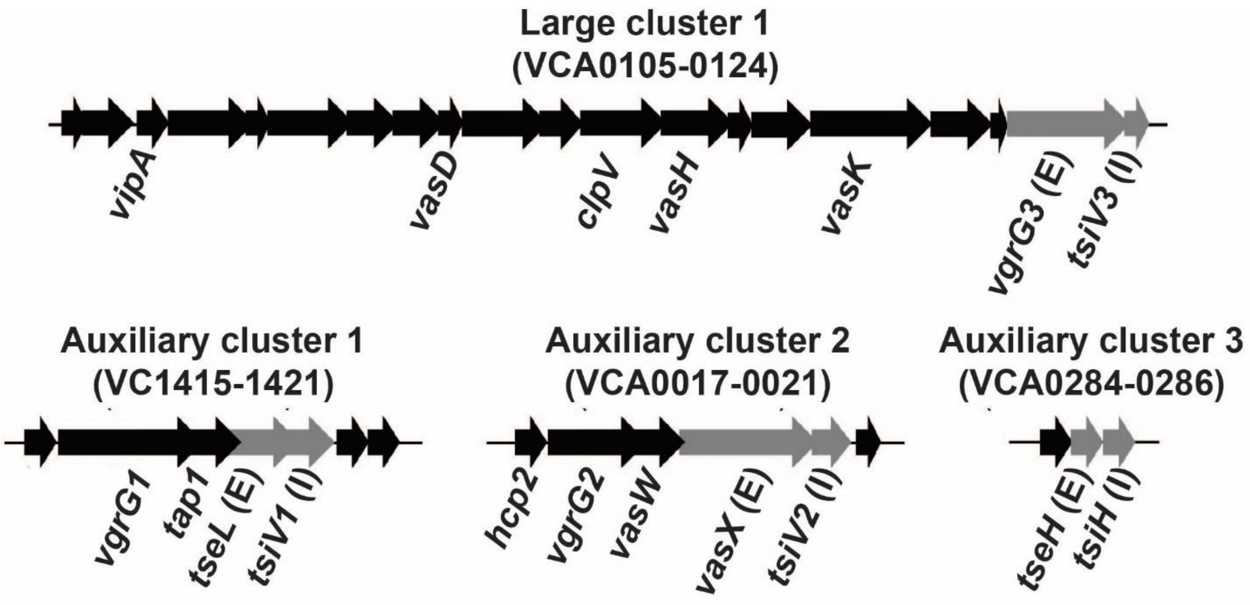

Supplementary Figure 1. Arrangement of T6SS genes in *V. cholerae* 2740-80. Select gene names are provided. Genes encoding T6SS effector and immunity proteins are depicted in gray and designated with, respectively, an E or I in parentheses. The large *t6ss* gene cluster is located on the major chromosome and the three auxiliary *t6ss* gene clusters are on the minor chromosome. The large cluster encodes the proteins that make the T6SS secretion complex and one effector-immunity protein pair. Each of the auxiliary clusters encodes one effector-immunity protein pair, among other genes. The figure was adapted from Metzeger et. al. 2016 and Mashruwala and Bassler 2022 (16, 37).

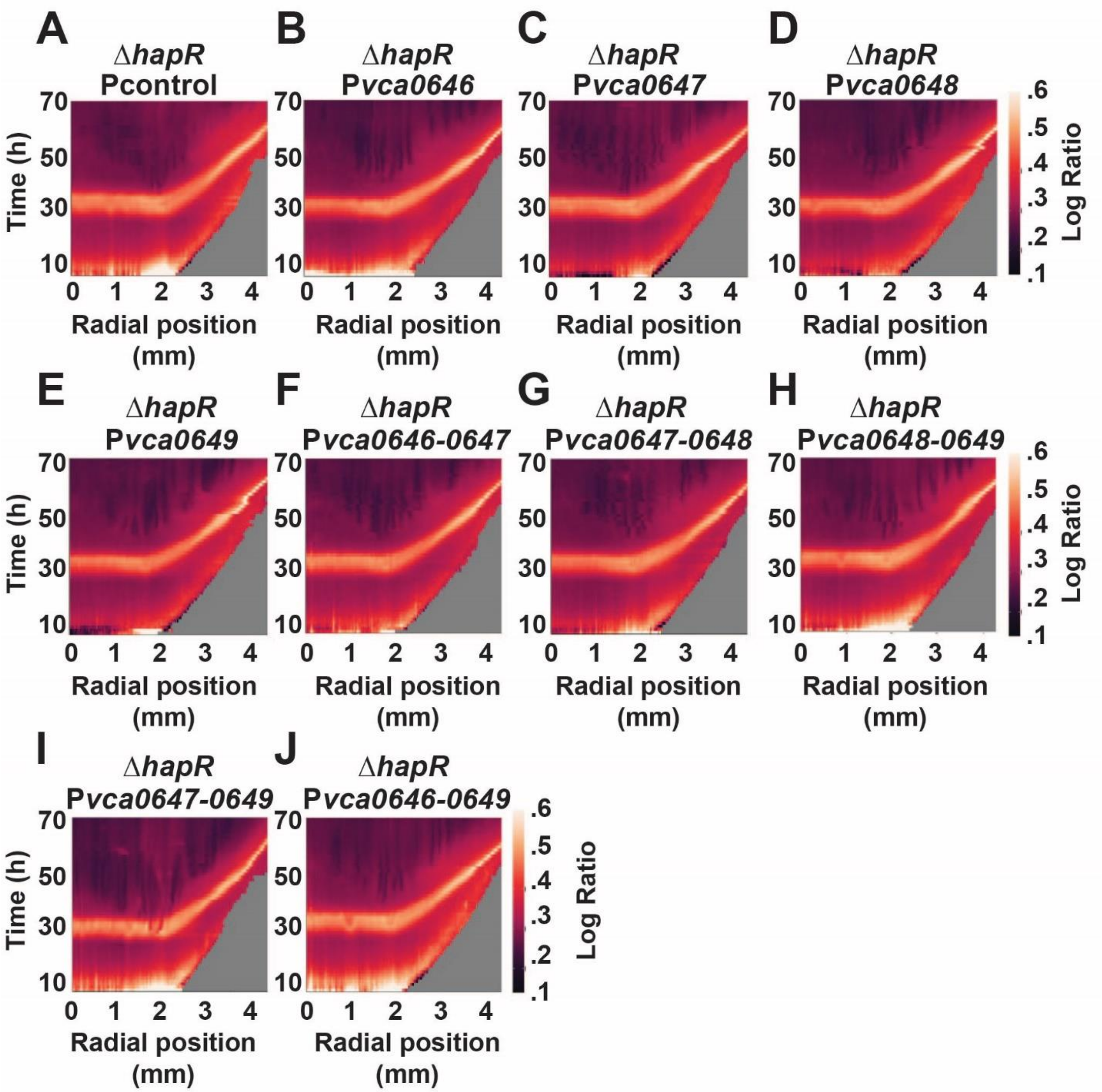

Supplementary Figure 2: Kymographs showing plasmid controls have no effect on rim cell death in the *V. cholerae* 2740-80 Δ*hapR* strain. (A–J) Logarithmic space-time kymographs showing cell death for the Δ*hapR* strain carrying the designated plasmids. All strains were cultured in the absence of arabinose. Kymographs from one colony are presented and are representative of results from ~3 colonies for each strain. These data are control kymographs for the same strains presented in Figure 6.

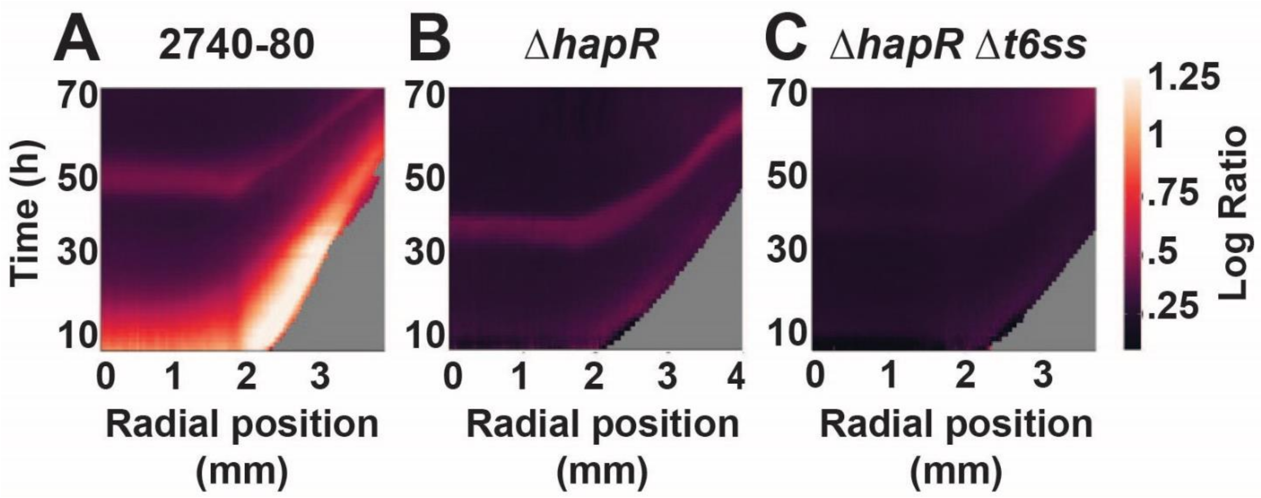

Supplementary Figure 3: Cell death at the centers of WT *V. cholerae* 2740-80 and Δ*hapR* colonies is driven by T6SS. (A–C) Logarithmic space-time kymographs showing cell death for the designated strains. Kymographs from one colony are presented and are representative of results from ~3 colonies for each strain. These data accompany Figure 7.

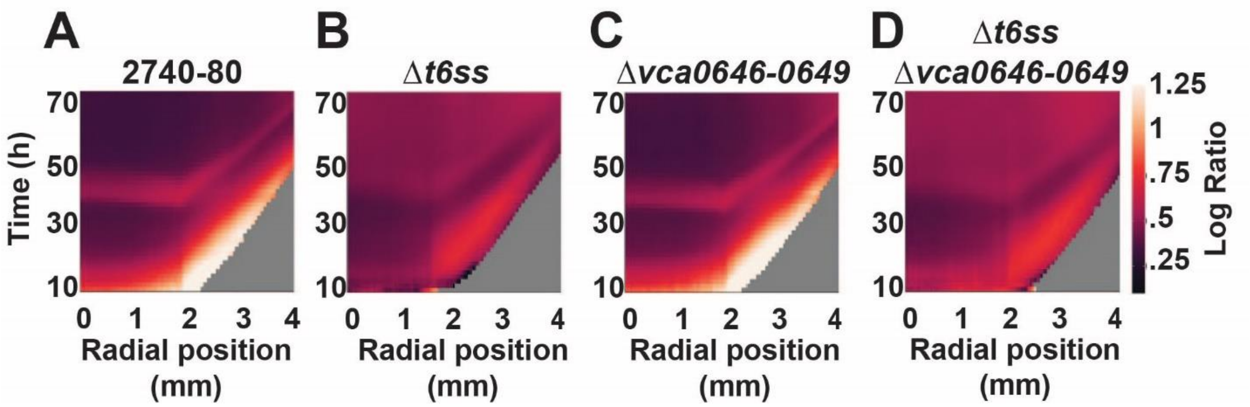

Supplementary Figure 4: Deletion of *vca646-649* does not alter the *V. cholerae* rim cell death pattern. (A–D) Logarithmic space-time kymographs showing cell death for the designated strains. Kymographs from one colony are presented and are representative of results from ~3 colonies for each strain.

**Table S1. Genotypes and phenotypes of QS strains and *hapR* variants used in this study. Data for p*luxC-lux* and p*vpsL-lux* were reported previously and are presented for ease of comparison** (1)**.**						
**Genotype**	**Type of mutation**	**p*luxC-lux* transcription: Activated by HapR at HCD. Levels relative to parent.**	**p*vpsL-lux* transcription: Repressed by HapR at HCD. Levels relative to parent.**	**p*hcp2-lux* transcription: Activated by HapR at HCD. Levels relative to parent.**	**T6SS killing: Levels relative to parent.**	**Cell death: Levels relative to parent.**
luxO A97E	Gain-of-function	Lower	Higher	Lower	Lower	Low rim cell death
Δ*hapR*	Loss-of-function	Lower	Higher	Same as parent	Same as parent	Low rim cell death
hapR A52T	Modification-of-function	Same as parent	Higher	Same as parent	Same as parent	Low rim cell death
hapR R123P	Attenuation-of-function	Lower	Higher	Same as parent	Same as parent	Low rim cell death
hapR 2aa insertion (after 54th aa)	Loss-of-function	Lower	Higher	Same as parent	Same as parent	Low rim cell death
hapR ORF interrupted by IS200/IS605-like element	Loss-of-function	Lower	Higher	Same as parent	Same as parent	Low rim cell death

**Table S2. Strains used in this study**			
**Strains**	**Genotype/Description**	**Genetic Background**	**Source/Reference**
AM-890	*lacZ*::P*tac-mKO*	2740-80	(1)
AM-933	*lacZ*::P*tac-mKO luxO* A97E	2740-80	(1)
AM-1169	*lacZ*::P*tac-mKO hapR* A52T; reconstructed variant	2740-80	This work
AM-1170	*lacZ*::P*tac-mKO hapR* R120P; reconstructed variant	2740-80	This work
AM-1171	*lacZ*::P*tac-mKO hapR* 2aa insertion; reconstructed variant	2740-80	This work
AM-1172	*lacZ*::P*tac-mKO hapR* IS200/IS605-like element insertion; reconstructed variant	2740-80	This work
AM-1130	*lacZ*::P*tac-mKO* Δ*vasK*	2740-80	(1)
AM-907	*lacZ*::P*tac-mKO* Δ*vpsL*	2740-80	(1)
AM-913	*lacZ*::P*tac-mKO* Δ*hapR*	2740-80	This work
AM-1087	*lacZ*::P*tac-mKO* Δ*hapR* Δ*vpsL*	2740-80	This work
AM-1229	*lacZ*::P*tac-mKO* Δ*vca646-0649*	2740-80	This work
AM-1027	*lacZ*::P*tac-mKO* Δ*vasK* Δ*vgrG3-tsiV3* Δ*vasX-tsiV2* Δ*tseH-tsiH* Δ*tseL-tsiV1* (Δ*t6ss)*	2740-80	(1)

AM-1232	*lacZ*::P*tac-mKO* ∆*vasK* ∆*vgrG3-tsiV3* ∆*vasX-tsiV2* ∆*tseH-tsiH* ∆*tseL-tsiV1* ∆*vca0646-0649 (*∆*t6ss* ∆*0646-0649)*	2740-80	This work
AM421	*Escherichia coli*	Top10	Bassler Lab Collection

**Table S3. Plasmids used in this study**		
**Plasmid name**	**Insert Locus/function**	**Source/Reference**
pKAS32	Suicide vector	Bassler Lab Collection
pKAS32-*vpsL*	Construct used to make chromosomal deletion	Bassler Lab Collection
pKAS32-*hapR*	Construct used to make chromosomal deletion	Bassler Lab Collection
pEVS-pBAD	Cloning vector for arabinose inducible gene expression	Bassler Lab Collection
pEVS-pBAD-*vca0646*	Overexpression construct	This work
pEVS-pBAD-*vca0647*	Overexpression construct	This work
pEVS-pBAD-*vca0648*	Overexpression construct	This work
pEVS-pBAD-*vca0649*	Overexpression construct	This work
pEVS-pBAD-*vca0646-47*	Overexpression construct	This work
pEVS-pBAD-*vca0647-48*	Overexpression construct	This work
pEVS-pBAD-*vca0648-49*	Overexpression construct	This work
pEVS-pBAD-*vca0647-49*	Overexpression construct	This work
pEVS-pBAD-*vca0646-49*	Overexpression construct	This work
P*hcp2-luxCDABE*	Luciferase-based *hcp2* transcriptional reporter	This work
Ptac-*qrr*4	Plasmid for overexpression of *qrr*4 under control of the Ptac promoter	Bassler Lab Collection

Reference:

1. A. A. Mashruwala, B. Qin, B. L. Bassler, Quorum-sensing- and type VI secretion-mediated spatiotemporal cell death drives genetic diversity in *Vibrio cholerae*. *Cell* S0092-8674(22)01125–4 (2022). https://doi.org/10.1016/j.cell.2022.09.003.
